# Effects of Macro Fibers on Crack Opening Reduction in Fiber Reinforced Concrete Overlays

**DOI:** 10.3390/polym16162282

**Published:** 2024-08-12

**Authors:** Sanghwan Cho, Amanda C. Bordelon, Min Ook Kim

**Affiliations:** 1 Department of Civil Engineering, Seoul National University of Science and Technology, 232 Gongneung-ro, Nowon-gu, Seoul 01811, Republic of Korea; sanghc9812@naver.com; 2 Mechanical and Civil Engineering Department, Utah Valley University, 800 West University Parkway, Orem, UT 84058, USA; amanda.bordelon@uvu.edu

**Keywords:** macro fibers, crack opening width, fiber-reinforced concrete, concrete overlays, pavement design

## Abstract

Macro fibers have been extensively used in the construction of various concrete structures, including bridges, dams, tunnels, industrial floors, and pavements. However, their effectiveness in reducing crack opening widths in concrete pavements has not been fully explored. This study aims to delineate the role of fibers by identifying the optimal types and volumes for effectively controlling cracks in concrete pavement structures, particularly in thin overlays. The research investigates how different fiber types, such as synthetic and steel, and their respective volumes can mitigate crack propagation in concrete overlays. Additionally, it evaluates the performance of fiber-reinforced concrete overlays compared to conventional dowel bar systems in terms of crack width reduction and overall pavement durability. The findings aim to provide specific design criteria for incorporating macro fibers in concrete overlays to enhance structural integrity and longevity.

## 1. Introduction

Since the 1960s, fibers have been extensively used in the construction of various concrete structures, including bridges, dams, tunnels, floors, and pavements [1,2,3,4]. The first reported type of fiber was steel, used primarily for reinforcement purposes [4]. Fiber-reinforced concrete (FRC) is widely utilized to enhance not only the performance of concrete but also new infrastructure projects and in the maintenance, repair, and strengthening of existing structures. This widespread adoption is driven by the significant advantages fibers offer in controlling concrete cracking. In fiber-reinforced cementitious composites, fibers serve to bridge developing cracks. Their effectiveness varies based on the type, volume, and configuration of the fibers.

Numerous studies have been conducted to elucidate the roles of different fibers and optimize their type and volume to meet the specific requirements of concrete structures [5,6,7,8,9,10,11,12,13,14,15,16,17,18,19,20]. Wen et al. investigated the effect of fibers on the mechanical properties and durability of ultra-high-performance concrete (UHPC) and confirmed that the optimal fiber volume fraction is more closely related to the fiber type than to the fiber aspect ratio [5]. Hosseinzadeh et al. compared three different fibers—steel, polypropylene (PP), and high-performance PP (HPP)—focusing on mechanical properties and durability [6]. They confirmed that HPP fibers exhibited improved flexural and tensile strengths compared to steel under the same loading condition. The effect of steel fiber incorporation on concrete fracture properties and durability was investigated [7,8,9]. It was reported that the fracture properties of steel fiber-reinforced concrete (SFRC) improved significantly with the increase in randomly distributed steel fiber volume fractions from 0 to 1.6% [7]. Rocha et al. conducted both short- and long-term experiments to clarify the pullout behavior of macro synthetic fibers, reporting that fiber configuration, surface conditions, and elastic modulus all play significant roles in the bond between the fiber and cement matrix [10]. Zainal et al. confirmed the hybrid effects of combining micro and macro synthetic fibers in improving the load-carrying capacity of hybrid fiber-reinforced concrete slabs [11]. The effects of basalt fiber on the mechanical properties and durability of concrete were examined, and improvements in durability, load-bearing capacity, and ductility were reported [12,13]. The advantages of using jute fiber in concrete for reinforcement purposes were recently highlighted, including improved strength and the potential to fill microcracks [14]. One of the recent research trends in fiber reinforcement is the recycling of fibers themselves or adding fibers to recycled concrete for green construction [15,16,17]. Furthermore, the hybrid effect is also important for better utilizing various fibers to enhance concrete durability or structural performance of concrete structures [18,19]. Cecconello and Poletto identified that using graphene oxide as a surface treatment for fibers can reduce voids at the fiber-matrix interface and decrease water interaction in the mixtures, thereby enhancing the durability of the concrete [20].

Adding fibers to concrete pavements enhances toughness, durability, and crack resistance, enabling structures to withstand repetitive vehicle traffic and environmental stresses, such as temperature fluctuations and moisture. Various types of fibers, each with unique properties, contribute to these objectives. Steel fibers, known for their high tensile strength and ductility, significantly improve load-bearing capacity and crack resistance, effectively controlling crack widths and distributing loads [21,22,23]. This makes them ideal for heavy-duty pavements. PP fibers help reduce shrinkage and thermal cracking, though they do not significantly contribute to the structural capacity of the pavement [24,25,26,27]. Alkali-resistant glass fibers enhance both the tensile strength and flexural capacity, but their use requires careful selection to avoid alkali–silica reactions [28,29]. Basalt fibers offer a good balance of strength, chemical resistance, and thermal stability, suitable for environments prone to corrosion or chemical degradation [30,31]. While steel fibers are generally recommended to extend the lifespan of structures under repetitive stress, the choice of fiber type and volume should be tailored to specific project needs, considering traffic load, environmental conditions, and budget constraints. For cost-sensitive projects, PP fibers are a viable option, whereas steel fibers are preferred for high-performance applications that require enhanced load distribution and crack control [32,33]. Increasingly, hybrid applications that combine different fiber types are being used to maximize the benefits [34]. Key design considerations to extend the service life of concrete overlays might include determining the appropriate thickness, joint design, subgrade preparation, and material selection. A well-designed concrete overlay must balance these factors to ensure a durable, cost-effective, and functional structure that ensures user safety and withstands environmental and operational stresses. Concrete overlays can exhibit significant crack spacing and wide crack openings when exposed to repeated high-temperature variations. Previous studies have demonstrated that incorporating fibers into concrete can effectively mitigate these crack openings [35,36]. However, the performance of FRC pavements can vary based on the type of fiber, volume content, surface geometry, and aspect ratio [37]. Research on FRC overlays indicates that crack width is primarily influenced by the extent of debonding and the fibers’ ability to bridge cracks [38]. Further research is needed to quantify the effectiveness of fibers in reducing crack openings and optimize their type and content for pavement structures.

**Figure 1:**
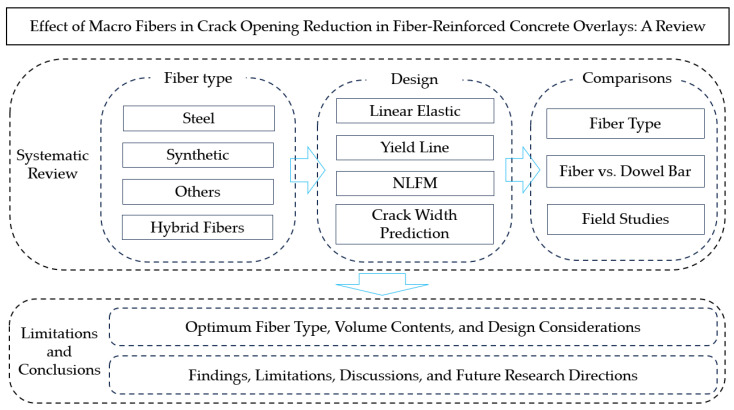
Research flow adopted in this study.

This study examines the impact of fiber addition on reducing crack openings in concrete pavement, drawing from a comprehensive review of the existing literature. Figure 1 illustrates the research flow and highlights the section keywords focused on in this study. The review aims to elucidate the optimal type and volume of fibers, identify critical design considerations for incorporating macro fibers in concrete pavements, and suggest limitations and directions for future research. It is important to note that field studies are essential for validating the effects of fiber integration in real-world concrete structures. Additionally, the efficiency of fiber addition in minimizing crack widths was reassessed by comparing it with the performance of traditional dowel bars. This comparison helps underscore potential improvements in pavement durability and effectiveness. The review also reevaluated current design guidelines for FRC overlays, highlighting essential design factors that require consideration. This research can contribute to an ongoing project aimed at integrating macro fibers into slabs on large concrete structures, primarily for energy storage applications. The findings from this study are expected to enhance the structural integrity and longevity of such concrete infrastructures.

## 2. Fiber Reinforcements for Pavement Structures

Table 1 shows the mechanical properties of representative fibers that can be applied to FRC overlays [12,39,40,41,42,43,44,45,46,47,48,49,50,51,52,53,54,55,56]. It can be observed that the tensile strength of steel fiber is approximately twice as high as that of PP fibers. Determining the optimal fiber type and volume for concrete pavements involves considering various factors such as the desired material properties, anticipated environmental conditions, and cost-effectiveness. The choice of fiber reinforcement also depends on the specific design requirements of each project. In the following section, we summarize the effects of selected representative fibers on potential applications in pavement structures. Additionally, Table 2 presents the effect of fiber reinforcements on strength improvement and thickness reduction in concrete pavements [28,29,30,31,32,33,34,35,36,37,38,39,40,49,50,51,52,53,54,55,57,58].

It can be seen that incorporating fiber reinforcement into concrete pavements can contribute to increased strength and reduced thickness, although the primary purpose of using fibers in conventional concrete is not to enhance strength. Furthermore, adding more than 2.0% fiber volume fraction may have a negative effect on strength [65,66,67]. The following summarizes the main characteristics of each type of fiber when incorporated into concrete pavements, including overlays.

### 2.1. Steel

Steel fiber has been extensively studied and used in pavement applications, as evidenced by numerous studies [28,29,33,35,38,68,69,70]. Hussain et al. [28] compared the effects of steel, PP, and glass fibers on the thickness reduction and strength enhancement of FRC pavements, finding that steel fibers were superior in both aspects, although more costly. Similarly, Ali et al. [29] observed significant improvements in mechanical properties such as flexural and residual strengths in FRC with hooked steel fibers, with increases ranging from 10 to 26% in flexural strength and 30 to 157% in residual strength. Achilleos et al. [33] advocated for the use of steel fibers in concrete pavement based on life cycle cost (LCC) analysis and life cycle assessment (LCA) results. Destrée et al. [68] demonstrated the effectiveness of steel fibers in controlling crack formation in concrete slabs through field tests and finite element modeling, highlighting the impact of fiber volume fraction, friction coefficient, and bond strength on crack control. Lau et al. [69] investigated the fatigue performance of FRC with 35 mm long end-hooked steel fibers in thin rigid pavements, noting a significant increase in fatigue resistance of over 100%. They suggested that combining steel fibers with conventional steel rebars could further enhance pavement durability under fatigue loading. Finally, Chen et al. [70] reported that adding 1.5% steel fiber to concrete significantly improves resistance to wheel impact, particularly under conditions of elevated temperature aging.

### 2.2. Synthetic

Nobili et al. [26] conducted a detailed examination of integrating PP fibers with an aspect ratio of 50 into concrete pavements in tunnel environments, revealing significant enhancements in structural performance. Pakravan and Ozbakkaloglu [71] observed that adding PP fibers to cementitious composites notably increases ductility and flexural toughness. Chen et al. [72] assessed the durability of four different synthetic fibers—polyester, monofilament PP, reticular PP, and polyacrylonitrile—in airport pavements, finding that polyacrylonitrile exhibited superior impermeability, while polyester was most effective against frost. They concluded that the optimal volume fraction for these fibers ranges between 0.10% and 0.14%. Merhej et al. [73] reported a 27% improvement in the modulus of rupture with twisted PP fibers at a 0.6% volume fraction. Hasani et al. [74] explored the effects of modified PP fibers (46.7 mm in length) on the mechanical properties and durability of FRC overlays, noting improvements in strength and ductility, along with a reduction in overlay thickness, although freeze–thaw resistance slightly decreased. Barman and Hansen [75] demonstrated that synthetic fibers improve a pavement’s load transfer efficiency and reduce differential displacement and joint energy dissipation, as evidenced by large-scale experiments and actual FRC overlay comparisons. Roesler et al. [76] confirmed that adding 40 mm long macro synthetic fibers (PP and PE) within a volume range of 0.32% to 0.48% enhances the structural behavior of FRC slabs, with tests showing at least a 20% increase in both flexural strength and ultimate cracking loads. Ali et al. [77] found that incorporating 0.2% PP fiber by weight into runway pavements enhances their impact resistance. Al-Rousan et al. [78] found that incorporating 0.90% PP fiber by volume significantly enhances the structural integrity and impact resistance of concrete slabs, although increasing the dosage to 1.2% did not offer additional benefits. Regarding concrete shrinkage, Choi et al. [79] confirmed a substantial reduction in both drying and autogenous shrinkage with the addition of 0.2% nylon fibers, suggesting potential applications in FRC pavement structures. Folliard and Berke [80] observed that plastic shrinkage cracking could be reduced by adding 0.1% of PP or nylon fibers by volume. Additionally, Gryzbowski and Shah [81] noted that fiber additions could reduce crack opening widths associated with drying shrinkage. Conversely, Wang et al. [82] reported minor or negative effects from adding polyoxymethylene fibers to concrete, based on mechanical properties and fatigue performance comparisons with a control group. Overall, while synthetic fibers enhance the durability and toughness of concrete pavements, they also pose challenges related to cost, handling, and finishing. Innovations in fiber geometry and surface design can bring improved adhesion to the cement matrix. High-strength PP fibers, particularly when combined with high-modulus fibers like steel, have proven effective in reinforcing concrete and enhancing its ductility.

### 2.3. Other Fibers

The optimal basalt fiber content for road construction was determined to be approximately 2.0% by weight, which significantly enhanced the mechanical properties reported [31]. Specifically, experiments showed a 20% increase in compressive strength, a 20% to 25% increase in tensile strength in bending, and a 15% to 20% improvement in frost and water resistance. Conversely, Sarkar and Hajihosseini [56] observed that incorporating basalt fibers into FRC pavements made them more brittle and reduced cracking resistance, compared to pavements reinforced with alternative fibers. Banthia et al. [83] noted that micro cellulose fibers (up to 0.3% by volume) effectively prevented cracking and reduced slab curling under continuous heat and moisture conditions. Khan and Ali [84] reported that using human hair fibers or wave-shaped PP fibers not only improved mechanical properties but also enabled a reduction in slab thickness to 12.5 mm, potentially saving up to 3% in construction costs.

### 2.4. Hybrid Fibers

Ozturk and Ozyurt [32] conducted a comprehensive study using experimental and numerical methods to assess the effectiveness of combining macro steel and PP fibers in reinforced concrete (RC) pavements, demonstrating significant reductions in thickness and improvements in post-cracking performance. Similarly, Shakir et al. [34] observed that hybrid fibers substantially decrease cracking and enhance structural performance in FRC pavements, a finding supported by extensive literature analysis. Yu et al. [30] reported that integrating both steel and basalt fibers into cementitious composites significantly enhances resistance to freeze–thaw cycles due to improved interfacial adhesion. Additionally, a synergistic effect of steel and nylon fibers was observed in reducing autogenous shrinkage, with 0.2% fiber content proving more effective than 0.3% [79].

Steel fibers, effective in controlling crack propagation, are preferred for heavy-duty pavements that require high load-bearing capacity and impact resistance. Bolat et al. [85] highlighted that SFRC exhibits superior mechanical properties and abrasion resistance compared to PFRC with PE or PP fibers. However, while steel fibers offer numerous benefits, they are costlier and may roughen surface finishes. Conversely, PP fibers, which help mitigate plastic shrinkage cracking and enhance durability, are lightweight, chemical-resistant, and non-corrosive, albeit offering less strength than steel fibers. Nevertheless, PP fibers significantly improve concrete toughness and provide a cost-effective solution for various applications. The subsequent sections will systematically compare the impacts of these fibers on crack reduction in concrete pavement structures, including overlays.

## 3. Design of FRC Overlays and Crack Opening Width Prediction

### 3.1. Design Methods Applicable to FRC Overlays

Table 3 outlines three design and analytical methods applicable to the design of FRC overlays [86,87,88,89,90]. The elastic response approach has traditionally been used for concrete pavements, assuming an infinitely thin concrete slab resting on an elastic foundation that remains in constant contact with the subgrade and presupposes a circular contact area for the wheel load [86]. However, this method cannot effectively capture the role of fibers, particularly in cases of nonlinear crack opening. Alternatively, the limit analysis method based on yield line theory, initially proposed by Johansen [87] and later endorsed by ACI and Meda et al. [88,89], offers a more precise design by focusing on the ultimate load. Specifically, yield line theory predicts the ultimate load-bearing capacity of concrete slabs, focusing on the plastic deformation along predefined ‘yield lines’ where the slab is expected to fail. This method is advantageous for providing a clear estimate of the ultimate load capacity and identifying potential failure mechanisms, which is crucial for safety. However, it does not consider the material behavior beyond initial yielding, which can limit its application in scenarios where post-cracking performance is critical. It also introduces challenges, such as addressing fatigue, temperature curling stresses, and traffic wander within a yield line framework, necessitating adjustments in elastic design procedures to account for the enhanced flexural capacity of FRC overlays.

Nonlinear fracture mechanics (NLFM) analysis provides another approach to examining the post-cracking behavior of FRC, particularly suited for strain-softening materials [90]. NLFM provides a detailed prediction of how cracks initiate, propagate, and affect the pavement over time, taking full advantage of the fibers’ properties to enhance durability and resilience. Despite its complexity and the need for detailed material data, NLFM offers a more comprehensive understanding of long-term pavement performance, crucial for ensuring durability and functionality. NLFM is also beneficial for the design of FRC overlays by analyzing thermal stress distribution, deformation, and the effects of dowel bars [91,92,93,94,95]. Masad et al. [91] used a 3D finite element model with interface elements to simulate joint interaction, demonstrating how uniform temperature changes affect joint openings and load transfer efficiency. Shoukry et al. developed a model to analyze thermal stresses in concrete pavements, considering the deformation constraints of dowel bars at transverse joints [92,93,94]. Mackiewicz et al. [95] explored the impact of various dowel bar diameters, showing that smaller diameters increase stress concentrations and induce tensile stress in the concrete adjacent to the bars. In essence, while the yield line theory provides essential insights into the structural safety and ultimate capacity of FRC pavements, NLFM offers a deeper understanding of the long-term behavior and effectiveness of fiber reinforcement, making it invaluable for designing durable and resilient pavement structures.

### 3.2. Residual Strength Ratio

The design of FRC overlays utilizes a residual strength ratio, *R_150_,* measured by the post-cracking flexural stress of the FRC and normalized by its flexural strength at first cracking, *MOR*. Regardless of when a concrete pavement would crack as a function of the *MOR*, a constant *R_150_* would indicate that a cracked overlay is still fundamentally resistant to the loading despite a reduced *R_150_* value from the increased *MOR*. In this regard, constant or increased residual strengths versus age would be expected for an FRC mixture, regardless of the use of the residual strength ratio in the current design methodology. Altoubat et al. [96] proposed the following equations to determine the effective modulus of rupture, *MOR_eff_* for the design of FRC overlays.

(1) $${MOR}_{eff}=MOR(1+R_{150})$$

(2) $${SR}_{total}=\frac{\sigma_{total}}{{MOR}_{eff}}$$

*R_150_* can be zero in conventional concrete pavement design, and the stress ratio, *SR_total_,* is estimated by dividing the total tensile stress from traffic and environmental loading, *σ_total_* by *MOR_eff_*. *R_150_* can be estimated following ASCE C1609 or JSCE-SF4 standards [97,98]. According to ASTM C1609 [97], both the residual stress, *f_L/150_,* and *R_150_* can be calculated using the following equations.

(3) $$f_{L/150}=P_{L/150}\cdot \frac{L}{bh^{2}}$$

(4) $$R_{150}=\frac{f_{L/150}}{MOR\cdot L/bh^{2}}\times 100$$

JSCE-SF4 [98] estimates *f_L/150_* and *R_150_* based on the toughness, *T_L/150_,* which represents the area under the load–deflection curve from 0 to 3.0 mm deflection.

(5) $$T_{L/150}=area{\left(P\cdot \delta \right)}_{0}^{L/150}$$

(6) $$f_{L/150}=\frac{T_{L/150}}{L/150}\cdot \frac{L}{bh^{2}}$$

(7) $$R_{150}=\frac{f_{L/150}}{MOR}\times 100$$

Unnotched flexural beam specimens are used to determine *R_150_* and *MOR* values. Moreover, various standardized empirical tests have been designed to accurately replicate real-world scenarios. For example, the ASTM C1550 [99] test evaluates a thin, circular panel under central loading to simulate point load cracking. Similarly, the ASTM C1609 [97] and JSCE-SF4 [98] tests assess the flexural strength and toughness of an unnotched beam under third-point or center-point loading. The ASTM C1399 [100] test measures residual strength and energy dispersal by loading an unnotched beam against a rigid plate, aiming to simulate distributed cracking. Each testing method is specifically designed to mimic or predict FRC behavior under particular loading conditions, typically progressing to a predetermined deflection or displacement level that correlates with the anticipated extent of cracking for the tested load. For instance, both the ASTM C1609 [97] and JSCE-SF4 [98] standards analyze the area under the load–deflection curve using third-point bending beams measuring 150 × 150 × 500 mm (with a span of 450 mm), up to a deflection of 3.0 mm.

### 3.3. Crack Opening Width Prediction in FRC Overlays

Table 4 summarizes the equations for estimating crack opening width in concrete overlays, including FRC overlays [101,102,103,104,105,106,107]. Equation No. 1 [101] in Table 4 estimates the crack width in continuously reinforced concrete pavement (CRCP) based on crack spacing, drying shrinkage, and concrete thermal expansion (CTE). It indicates that the crack width increases with greater crack spacing, which is also related to concrete tensile stress. Darter and Barenberg [102] developed Equation No. 2 in Table 4 to predict the joint opening width in joint plain concrete pavements (JPCP) based on CTE and drying shrinkage. This equation is also used in the AASHTO design guide [103]. Note that this equation is empirical as it does not consider the strain distribution in the slab and treats the friction factor as a unitless parameter, assuming uniform strain distribution throughout the slab. RILEM [104] suggested Equation No. 3 in Table 4, which modifies an equation originally proposed by ENV 1992-1-1 [105] to estimate the crack width in concrete structures. This modification considers the fiber aspect ratio (*L_f_*/*D_f_*) to quantify the effect of steel fibers in reducing the crack width. Löfgren [106] and Jansson et al. [107] proposed equations (listed as No. 4 in Table 4) to predict the crack width in SFRC beams, based on experimental results that include both *MOR* and *R_150_*. Overall, it can be concluded that a limited number of prediction equations are available, and further research is necessary.

## 4. Effect of Fiber Types on Crack Opening Reduction and Comparison of Macro Fibers to Dowel Bars

### 4.1. Effects of Fiber Types and Volume Contents on Crack Width Reduction in FRC Pavements

Table 5 summarizes the effects of fiber types and volume contents on reducing crack opening widths in FRC overlays [35,38,108,109,110,111,112,113,114,115,116]. It is evident that incorporating fibers effectively reduces further crack opening, though performance varies with fiber type and volume. Chanvillard et al. [35] confirmed significant reductions in the crack width with steel fibers in thin concrete overlays and reported an optimal content of 0.5% by volume, or 40 kg/m^3^. Carlswärd [38] reported narrower crack widths in 50 mm deep overlays that included steel fibers, emphasizing the crucial role of the interfacial bond between new and existing layers in influencing the crack width. The optimal amounts of steel and PP fibers were suggested to be higher than 20 kg/m^3^ and 2.5 kg/m^3^, respectively, to increase resistance to harsh acid attacks [57]. It was also highlighted that PP fibers offer a cost advantage compared to steel fibers, while both contribute similarly to strength improvement when dispersed in concrete pavement. Overall, both steel and PP fibers are effective in reducing crack opening widths, and hybrid fibers also show promise. However, field investigations are still lacking, and further studies are crucial to explore the long-term effects of fibers in FRC overlays.

### 4.2. Comparison of Macro Fibers to Dowel Bars

Dowel bars are employed primarily across transverse joints of concrete slabs to facilitate load transfer and maintain alignment [117]. These steel bars do not contribute directly to the internal reinforcement of the concrete but play a crucial role in preventing differential settlement at the joints [118]. While dowel bars do not address surface cracking within the concrete panels themselves, they are vital in ensuring the structural integrity and continuity of the pavement, particularly in areas subjected to heavy loads [119].

The efficiency of fibers in crack reduction is predominantly due to their ability to physically bridge cracks within the concrete and limit the development of visible cracks. This makes fibers particularly valuable in applications where surface integrity and reduced maintenance are priorities. In contrast, dowel bars are instrumental in enhancing the performance of pavement joints, which, although not directly influencing surface crack formation, are essential for the longevity and functionality of pavement structures under dynamic loads. Overall, fibers and dowel bars play important roles in pavement construction, each contributing uniquely to the structural integrity and performance of concrete slabs. While fibers enhance the concrete’s intrinsic properties by reducing surface cracks, dowel bars are crucial for maintaining slab alignment and facilitating effective load transfer across joints. This dual approach not only ensures a reduction in maintenance costs but also prolongs the lifespan of pavement structures.

## 5. Limitations and Future Studies

Macro synthetic fibers such as PP, PE, and PVA are increasingly integrated into concrete mixtures to enhance mechanical properties, notably improving flexural strength and post-cracking behavior. When added to concrete, these fibers act as a mesh, helping to bridge and manage the development of cracks that naturally occur over time. This bridging effect not only enhances the ductility and toughness of the concrete but also proves crucial in maintaining the structural integrity of pavement systems subjected to dynamic loads and environmental stressors. The refined formulation of FRC overlays demonstrates a significant reduction in crack formation and results in notably narrower cracks when they do occur. These improvements can greatly extend the service life of pavement structures by reducing maintenance demands and enhancing resistance to severe weather conditions and chemical degradation. Despite these benefits, the adoption of such fibers in concrete pavement construction faces significant challenges. The cost and accessibility of high-quality fibers can be prohibitive, potentially limiting their widespread use in infrastructure projects. Moreover, achieving an optimal concrete–fiber mix is critical to leveraging the full potential of this technology. The distribution and orientation of fibers within the mix are crucial as they significantly influence the final properties of the concrete. Achieving this requires precise engineering processes and rigorous quality control measures to ensure consistency across batches. Additionally, the long-term performance of FRC, while promising, remains less reported under real-world conditions. Over time, factors such as fiber degradation and interactions with environmental elements could impact the durability and functionality of FRC overlays.

The incorporation of PP fiber into concrete can present several drawbacks, including a diminished interfacial bond, reduced workability as evidenced by decreased slump values, and decreased compressive strength. These challenges underscore the need for comprehensive investigations to ensure the desired concrete properties and extend the operational lifespan of concrete pavement infrastructures. The strategic integration of polymeric modifiers such as ethylene-vinyl acetate (EVA), styrene-butadiene rubber (SBR), and epoxy resins shows promise in mitigating the identified shortcomings associated with concrete reinforcement. Previous research has demonstrated that a combination of polyester fibers at a volume of 0.14% and SBR latex at a concentration of 90 kg/m^3^ significantly enhances the mechanical characteristics of fiber-reinforced polymeric cementitious composites (FRPCC), as detailed by Xu et al. [120]. The presence of continuous SBR latex films within the cement matrix notably increases toughness and densifies the interface transition zone (ITZ), fostering a robust bond between polyester fibers and the cement paste.

**Figure 2:**
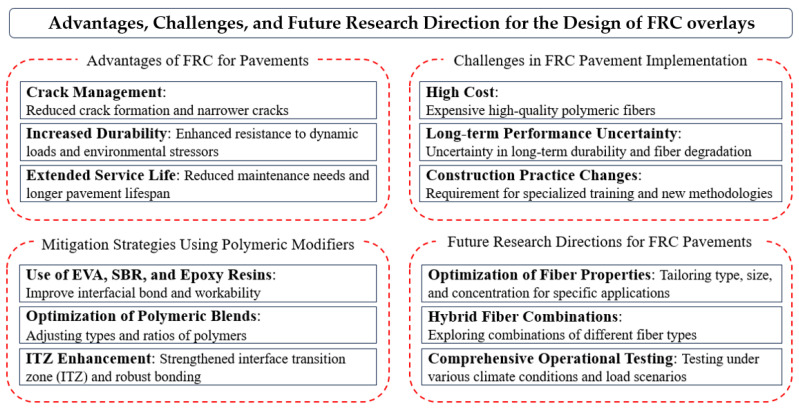
Limitations and future studies.

Looking forward, future research should focus on optimizing the type, size, shape, and concentration of fibers to maximize structural benefits while minimizing material costs, as shown in Figure 2. Studies on hybrid combinations of different fiber types could yield concrete mixes with tailored properties for specific applications. The environmental impact of producing and utilizing synthetic fibers in concrete also warrants further investigation. Life cycle assessments of FRC could provide deeper insights into its overall sustainability, highlighting areas for improvement. Furthermore, comprehensive testing of FRC under realistic operational conditions is essential to better predict the long-term behavior of FRC overlays and refine their composition and construction processes. Lastly, the development of new and innovative fiber materials, such as those derived from bio-based or recycled sources, represents a promising research domain. These materials could potentially offer comparable or superior performance to current synthetic fibers while aligning more closely with sustainability objectives.

While macro synthetic fibers present a valuable enhancement to concrete pavement technology, realizing their full potential requires overcoming several technical and practical challenges. Continued research and innovation in this field are essential to advance the knowledge base, optimize material properties, and facilitate broader adoption of this promising technology in pavement engineering.

## 6. Conclusions

This study aimed to delineate the role of fibers by identifying the optimal types and volumes to effectively control cracks in concrete pavement structures, especially in thin overlays. Based on the analysis and comparison, the following conclusions can be drawn:

1. Steel fibers demonstrate superior effectiveness in reducing the crack opening width compared to polypropylene (PP) fibers, offering significant durability and performance benefits for concrete overlays. However, cost considerations and potential corrosion issues must be carefully managed.
2. Hybrid systems combining macro and micro fibers exhibit excellent properties for reducing the crack opening width. Integrating different fiber types into hybrid systems is a promising strategy for improving both the structural performance and cost-effectiveness of concrete overlays. The combination of the high strength of macro fibers and the fine size of micro fibers maximizes the crack reduction effect, enhancing long-term performance.
3. Designing FRC overlays involves numerous complex variables beyond those of traditional overlay methods, including fiber type, volume content, and overlay thickness. With the proper design and material selection, FRC overlays can achieve outstanding performance.
4. Further field testing is necessary. Future research should assess the long-term performance and durability of concrete overlays with various fiber types and volume contents under realistic environmental conditions. Field test results will complement laboratory findings and bolster confidence in their real-world applications.

In addition, discussions of limitations and future studies were conducted to address potential weaknesses in the current research and guide further investigations.

## Figures and Tables

**Table 1:** Mechanical properties of representative macro fibers can be applied to FRC overlays [12,39,40,41,42,43,44,45,46,47,48,49,50,51,52,53,54,55,56].

Property	Fiber Type							
	Steel [39,40]	PP ^1 ^ [40,41,42,43]	PE ^2 ^ [39,44]	PVA ^3 ^ [45,46,47,48]	Polyester [49,50,51]	Polyolefin [52,53,54]	Nylon [42,55]	Basalt [12,56]
Specific gravity	7.84	0.91	0.92–0.96	1.20–1.30	1.33–1.40	0.91–0.97	1.10–1.16	2.52–2.97
Modulus of elasticity (GPa)	200	1.5–12	5–100	20–43	8–20	>9	4–5.3	85–110
Tensile strength (MPa)	500–2000	240–900	80–600	1000–1600	400–750	>500	450–919	1100–4840
Elongation at break (%)	0.5–3.5	15–80	4–100	6–7	12–20	15–30	15–28	3.15
Acid and Alkali Resistance	Varied	High	High	High	High	High	Moderate	High
Cost ($/kg)	1.0–8.0	1.0–2.5	2.0–20	1.0–15	1.2–1.5	1.0–10	2.0–2.5	4.5–5.0

^1^ PP: polypropylene; ^2^ PE: polyethylene; ^3^ PVA: polyvinyl alcohol.

**Table 2:** Effect of fiber reinforcement on strength improvement and thickness reduction in concrete pavements [28,29,30,54,57,58,59,60,61,62,63,64].

Fiber Type	Strength Improvement	Thickness Reduction and Other Effects	Refs.
Steel (Hooked or Wave)	Compressive strength 10% ↑ *, Flexural strength 80% ↑	Improved residual strength and toughness, Reduced thickness by 63 mm at 1.0% *V_f_* **	[28]
	Flexural strength 25% ↑ at 0.5% *V_f_* and 47% at 1.0% *V_f_*, Improved residual strength	Contributed to reduced thickness, Prevent micro cracking due to drying shrinkage	[29]
	Improved compressive and flexural strength	Improved cold, wear, and acid resistances	[57]
	Compressive strength 25% ↑ at 7 and 28 days	Increased ultimate load with the addition steel fibers and silica fume (confirmed through SEM *** & TGA ****)	[58]
PP	Tensile strength 20% ↑	Thickness reduced by 21mm at 1.0% *V_f_*	[28]
	Flexural strength 9% ↑ at 0.5% *V_f_* and 18% ↑ at 1.0% *V_f_*	Contributes to reduced thickness	[29]
	Compressive strength 5 to 6% ↑, Flexural strength 8 to 12% ↑	Improved wear and frost resistances (at 50 cycles)	[59]
PVA	Improved flexural and tensile strength values with increased *V_f_*	Prevents brittle failure of pavement in case of overload or subgrade support loss	[60]
	Improved compressive and flexural strength	Improved wear resistance by 44% and impact resistance more than doubled	[61]
Polyester	Improved strength compared to PP fibers	–	[62]
Polyolefin	Improved tensile strength	Reduced stress concentration and prevents counter cracks	[63]
	Similar strength and elastic modulus to unreinforced concrete Improved tensile strength	Improved load-bearing capacity	[54]
Nylon	Compressive strength 2.62 to 5.01% ↑ Flexural strength 12.31% ↑	Improved wear resistance (7.30%), Reduced permeability (37.5%)	[61]
Basalt	Highest compressive strength at 2.0% *V_f_*, Increased splitting tensile strength	Strength increased with the addition of kaolin or silica fume	[64]
Hybrid (Steel and Basalt)	Reduced compressive strength at freeze-thaw condition, but less compared to single fiber	Improved freeze-thaw resistance, increased pavement structure life	[30]
Hybrid (PP and Polyester)	Strength significantly increased compared to single fiber	–	[62]
Hybrid (PVA and Nylon)	Higher compressive and flexural strength compared to a single fiber	Suitable for emergency packaging repairs	[61]

* ↑ increasing; ** *V_f_*: fiber volume fraction; *** SEM: Scanning Electron Microscopy **** TGA: Thermogravimetric analysis.

**Table 3:** Methods for the design of concrete pavement including FRC overlays [86,87,88,89,90].

Feature	Linear Elastic		Non-Linear Fracture Mechanics
	Elastic Response	Yield Line	
Approach	Elastic foundation with constant subgrade contacts and a circular wheel load contact area	Based on yield line theory, focuses on ultimate load capacity	Analyzes post-cracking behavior, particularly for strain-softening materials
Based on	Westergaard stress formulation [86]	Yield line theory [87]	NLFM principles
Pros	Traditionally used for straightforward scenarios	Offering precise design calculations for ultimate load	Accurate, providing detailed predictions and enhances durability
Cons	Not fully capture the role of fibers, especially in crack openings	Requires adjustments for fatigue, temperature curling stresses	Complex and requires detailed material data
Limitation	Not useful for complex stress conditions or advanced material behaviors including fibers	Does not account for material behavior beyond initial yielding	Challenging to integrate into existing design frameworks
Other	Used primarily for initial design estimates	Modifications needed in elastic design procedures to account for FRC	Beneficial for analyzing thermal stress distribution, deformation, and the effects of dowel bars

**Table 4:** Existing equations to predict the crack opening width in concrete pavements [101,102,103,104,105,106,107].

No.	Equation	Symbols	Refs.
1	$cw=CC\cdot L\left(\varepsilon_{shr}+\alpha_{PCC}∆T-\frac{c_{2}f_{\sigma }}{E_{PCC}}\right)$ *cw* is 0, if *cw* is less than 0	*cw* = crack width at the depth of the steel, *CC* = local calibration constant (1 is recommended in MEPDG [83] based on global calibration), *L* = mean crack spacing, *ε_shr_* = drying shrinkage coefficient of Portland cement concrete (PCC), *α_PCC_* = coefficient of thermal expansion (CTE) of PCC, *ΔT* = drop in PCC temperature from the concrete set temperature at the depth of the steel, *c_2_* = second bond stress coefficient increment, *f_σ_* = maximum longitudinal tensile stress in PCC at the steel level, *E_PCC_* = elastic modulus of PCC	[101]
2	$∆L=CL(\alpha_{t}∆T+\varepsilon )$	*∆L* = joint opening width, *C* = adjustment factor (0.65 is a typical), *L* = joint spacing or slab length, *α_t_* = coefficient of thermal expansion, *∆T* = temperature differences at placement, *ε* = drying shrinkage coefficient	[102,103]
3	$w=\beta \varepsilon_{sm}\left(50+0.25k_{1}k_{2}\frac{\phi_{b}}{\rho_{r}}\right)\left(\frac{50}{L_{f}/D_{f}}\right)$	*w* = crack width, *β* = coefficient relating the average crack width to a structural design, *ε_sm_* = mean strain between the cracks in the tensile reinforcement, *k_1_* and *k_2_* = non-dimensional geometric coefficients, *ϕ_b_* = structural tensile reinforcement bar diameter, *ρ_r_* = tensile reinforcement ratio, *L_f_*/*D_f_* = fiber aspect ratio	[104,105]
4	$w=\left(\varepsilon_{sm}-\varepsilon_{cm}\right)\;\left\{3.4c+0.425k_{1}k_{2}k_{5}\frac{\phi }{\rho_{s,eff}}\;\right\}$ $k_{5}=\left(1-\frac{f_{residual}}{f_{ctm}}\right)$	*Ɛ_sm_* = mean strain in the structural tensile reinforcement, *Ɛ_cm_* = mean strain in the remaining concrete between the cracks, *c* = concrete cover depth, *k_1_* and *k_2_* = non-dimensional geometric coefficients, *f_residual_* = measured residual flexural stress of SFRC, *f_ctm_* = measured flexural strength of SFRC, *ϕ* = structural tensile reinforcement bar diameter, *ρ_s,eff_* = effective structural tensile reinforcement ratio	[106,107]

**Table 5:** Effects of fiber type and volume content on crack width reduction in FRC overlays [35,38,108,109,110,111,112,113,114,115,116].

Fiber Type	Fiber Volume, *V_f_*	Effect on Crack Opening Width Reduction	Refs.
Steel	0 to 1.0%	Restrain crack development, improve crack resistance, load transfer, and enhanced structural durability	[35]
	0.75%	Effectively limit crack opening width, well-distributed micro-cracks, contribute to high bond strength	[38]
	0 to 1.25%	Increased first cracking load with higher *V_f_*, The first cracking load increased by 21% at 1.25% *V_f_*	[108]
	0 to 0.75%	Improved cracking resistance and load transfer capacity with higher *V_f_*, enhanced durability	[109]
	0.1%	Initial crack width reduced by 50%, Crack width increased over time with signs of corrosion observed	[110]
	0.6 to 0.8%	Reduced crack opening width with increased *V_f_*	[111]
PP	0.1%	Reduced crack width by 84% and initial crack age increased by 62% No full-depth cracks observed after 28 days	[110]
	0 to 0.88%	Improved cracking resistance and load bearing capacity	[112]
Polyolefin	0.1%	Delayed initial crack age, but no significant effect on crack width	[110]
	0 to 0.88%	Contribute to better load recovery with maintaining greater load-carrying capacity	[112]
PVA	0.25 to 0.50%	Reduced crack width by 70% for macro fibers and 90% for micro fibers, shrinkage reducing admixtures (SRA) applied	[113]
Glass	0 to 10%	Reduced crack opening widths with increased *V_f_*	[114]
	0.125 to 0.75%	Reduce crack width, but promote multiple cracks, Effective at 0.25% *V_f_*	[115]
	0.1%	Delayed initial crack age, but no significant effect on crack width	[110]
Basalt	0.1%	Delayed initial crack age, but no significant effect on crack width	[110]
Hybrid (Steel and PP)	0.75% (Steel: 0 to 60 kg/m^3^) (PP: 0 to 6.8 kg/m^3^)	Hybrid fibers reduce crack width and enhance post-cracking behavior, with steel fibers increasing toughness and polypropylene fibers reducing variability	[116]

## Data Availability

The original contributions presented in the study are included in the article, and further inquiries can be directed to the corresponding authors.
